# Soybean (*Glycine max*) Is Able to Absorb, Metabolize and Accumulate Fenbendazole in All Organs Including Beans

**DOI:** 10.3390/ijms22136647

**Published:** 2021-06-22

**Authors:** Radka Podlipná, Martina Navrátilová, Lucie Raisová Stuchlíková, Kateřina Moťková, Lenka Langhansová, Lenka Skálová, Barbora Szotáková

**Affiliations:** 1 Laboratory of Plant Biotechnologies, Institute of Experimental Botany, Czech Academy of Sciences, 165 02 Prague, Czech Republic; podlipna@ueb.cas.cz (R.P.); motkova@ueb.cas.cz (K.M.); langhansova@ueb.cas.cz (L.L.); 2 Department of Biochemical Sciences, Faculty of Pharmacy, Charles University, 500 05 Hradec Králové, Czech Republic; navratimart@faf.cuni.cz (M.N.); stuchli2@faf.cuni.cz (L.R.S.); skaloval@faf.cuni.cz (L.S.)

**Keywords:** pharmaceuticals, anthelmintics, benzimidazoles, biotransformation, antioxidant enzymes, isoflavonoids

## Abstract

Although manure is an important source of minerals and organic compounds it represents a certain risk of spreading the veterinary drugs in the farmland and their permeation to human food. We tested the uptake of the anthelmintic drug fenbendazole (FBZ) by soybean, a common crop plant, from the soil and its biotransformation and accumulation in different soybean organs, including beans. Soybeans were cultivated in vitro or grown in a greenhouse in pots. FBZ was extensively metabolized in roots of in vitro seedlings, where sixteen metabolites were identified, and less in leaves, where only two metabolites were found. The soybeans in greenhouse absorbed FBZ by roots and translocated it to the leaves, pods, and beans. In roots, leaves, and pods two metabolites were identified. In beans, FBZ and one metabolite was found. FBZ exposure did not affect the plant fitness or yield, but reduced activities of some antioxidant enzymes and isoflavonoids content in the beans. In conclusion, manure or biosolids containing FBZ and its metabolites represent a significant risk of these pharmaceuticals entering food consumed by humans or animal feed. In addition, the presence of these drugs in plants can affect plant metabolism, including the production of isoflavonoids.

## 1. Introduction

Anthelmintic drugs are used to control parasitic worms in animals as well as in humans. The benzimidazoles represent the largest chemical group of anthelmintics which disrupt the cytoskeleton by selectively interacting with tubulin of parasites. Fenbendazole (FBZ), a broad-spectrum benzimidazole anthelmintic active against gastrointestinal parasites including giardia, roundworms, hookworms, whipworms, tapeworms, pinworms, paragonimiasis, strongyles, and strongyloides, is commonly used in the treatment of sheep, cattle, horses, fish, dogs, cats, rabbits, and seals. In human, FBZ is considered for the treatment of cancer and fungal infection [1,2]. The active metabolite of FBZ, oxfendazole, becomes a promising antiparasitic drug available to treat helminthic diseases of humans [3].

Although the usefulness and necessity of anthelmintics, including FBZ, is unquestionable, their widespread use leads to environmental contamination and might have harmful effects on non-target species due to the abundant excretion of parent substance and metabolites. Generally, control strategies have focused on egg suppression regimens that involve the frequent application of anthelmintics to individuals at intervals based on egg reappearance periods after treatment [4]. Anthelmintic drugs can be administered in a variety of ways, including orally, by injection, or skin spray. The deworming regime varies depending on the type of equipment and the species. For example, all herd can be treated at once, which leads to large but relatively rare “inputs” of high-intensity anthelmintics entering the environment, while some farmers treat animals in small groups, resulting in regular pulses at lower doses [5]. On the other hand, the use of long-acting or sustained-release methods (long-acting injection, slow-release boluses, or reticulorumen devices) usually result in the continuous excretion of low concentrations of residual anthelmintic [6].

It has been known for several decades that pharmaceuticals dispersed in the environment, even at low concentrations (ng/L), pose risk to aquatic life and human health [7]. In recent years, there has been a growing interest in the fate and behavior of veterinary medicinal products in manure and fertilized soils. While different types of veterinary drugs were tested intensively, the group of anthelmintics was out of interest for a long time. The first study dealing with the fate of FBZ in manure described the slow degradation of FBZ in pig manure: the storage of manure for 6 months did not lead to a significant reduction of FBZ environmental exposure [8]. In organisms, FBZ might exhibit a negative effect in non-target organisms [9,10], including plants. The possibility of plants to uptake, accumulate and biotransform the FBZ was described previously. FBZ was not only readily absorbed but even evoked stress in *Plantago lanceolata*, as it twice increased the concentration of the plant stress marker proline [11] or significantly affected gene and protein expression in *Arabidopsis thaliana* plants [12].

The information about the fate of anthelmintics in crops, fertilized with manure from treated animals, has been limited, although it is known that the presence of low concentrations of anthelmintics in plants used as animal feed or as food for humans can encourage the development of resistant parasite strains [13,14,15,16,17]. For this reason, we have focused on anthelmintics in soybeans, whose beans are processed into “Soybean Meal” and fed to livestock or are widely consumed in different products by people around the world [18]. We decided to test the biotransformation of benzimidazole anthelmintic FBZ in soybean and to monitor its accumulation, predominantly in beans. The effects of FBZ on the activities of antioxidant enzymes and the synthesis of polyphenols, especially isoflavones, were also studied.

## 2. Results and Discussion

Veterinary drugs have the potential to enter the environment, where they may affect non-target organisms including plants [19] and can also affect human and animal health. We described the uptake, biotransformation, and effects of benzimidazole anthelmintic FBZ in several plants: *Campanula rotundifolia*, *P. lanceolata*, and *A. thaliana* [11,12,19,20]. However, the information about FBZ fate in crop plants has been missing. Therefore, the present study was designed to test the uptake and accumulation of FBZ in soybeans (*G. max*, a common crop abundantly fertilized by manure). We sought to determine if FBZ present in manure from treated animals could eventually get into the beans consumed by humans or used as additive to cattle and sheep feed [18]. In addition, we monitored the effect of FBZ on plant growth, fitness, yield, antioxidant enzymes activities as well as isoflavones content to obtain more complex information about its impacts on crops.

Our results showed that the growth of soybean plants was not visibly affected by the application of FBZ. Also, the yield of beans in FBZ treated plants (number of seeds per plant 10.8 ± 1.2; seeds weight 1.73 ± 0.08 g) was the same as in control plants (number of seeds per plant 10.5 ± 1.6; seeds weight 1.79 ± 0.30 g).

### 2.1. Uptake and Metabolism of FBZ in Soybeans

FBZ biotransformation was studied previously in harebell (*C. rotundifolia*), ribwort (*P. lanceolata*), and in the model plant *A. thaliana*. It was shown that FBZ was extensively metabolized in plant cells by both Phase I (oxidation to FBZ S-oxide and FBZ sulphone, hydroxylation, and hydrolysis) and Phase II reactions (glycosylation and acetylation).

**Figure 1:**
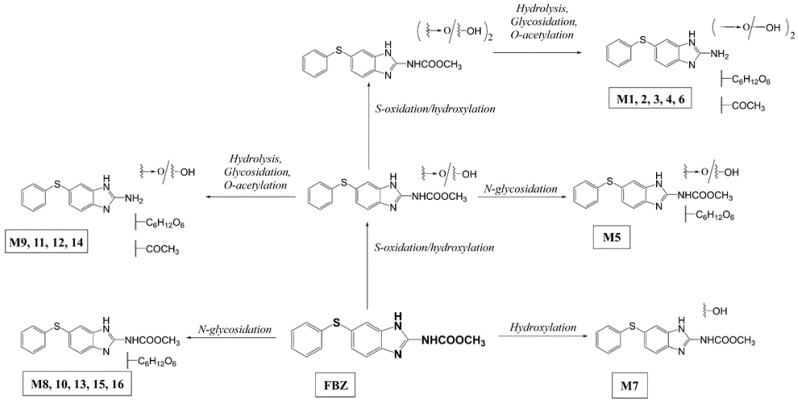
Biotransformation pathways of fenbendazole (FBZ) in soybean.

FBZ was extensively metabolized in the soybean plantlets as well. The metabolites were detected and identified using the UHPLC-MS/MS technique. All measurements were observed in the positive-ion mode. The detected metabolites were identified according to protonated molecules [M + H]^+^. The description of all FBZ metabolites (retention times, molecular weights, and fragment ions) detected in MS/MS experiments are summarized in Supplementary Table S1, the proposed scheme of FBZ metabolic pathways in soybean is shown in Figure 1.

FBZ was detected at *m*/*z* 300 [M + H]^+^ (t_R_ = 9.61 min.) with a product ion *m*/*z* 268 (typical neutral loss of methanol Δ*m*/*z* 32). Various oxidation, hydroxylation and hydrolysis reactions represented Phase I biotransformation reactions of FBZ. In Phase II, conjugation with UDP-hexose(s) predominated. The formation of some metabolites required a sequence of several reactions (two S-oxidations, hydrolysis, hydroxylation, glycosylation and acetylation). Five different FBZ *N*-glycosides (M8, M10, M13, M15, M16) and one oxidized FBZ *N*-glycoside (M5) were detected. The characteristic neutral loss of hexose, Δ*m*/*z* 162, was observed in MS/MS spectra of all these conjugates. Hydrolyzed FBZ acetylglycosides with one-step S-oxidation (M9, M11, M12, M14) and two-step S-oxidation (M1, M2, M3, M4, M6) both with neutral loss of Δ*m*/*z* 220 (*O*-acetyl-glycoside) were found. In the total of 16 FBZ metabolites were found in soybean plantlets and whole plants. As with other plant species, glycosylation is the most important biotransformation reaction of FBZ in *Glycine max*.

**Figure 2:**
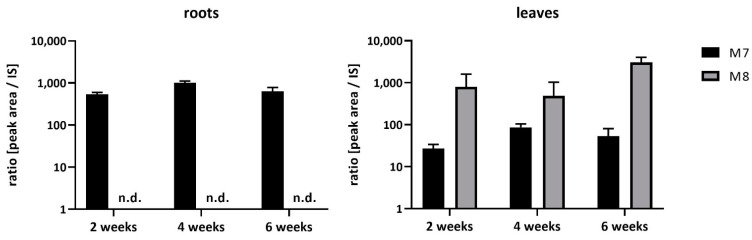
The time-dependent formation of FBZ main metabolites (M7 and M8) in soybean roots and leaves. Mean ± SD, n = 3; n.d. = not detected.

Each metabolite amount was semi-quantified using the ratio between peak area of metabolite and peak area of internal standard (IS). The peak area was normalized to 1 g of lyophilized plant samples. The most abundant FBZ metabolites found in soybean was hydroxylated FBZ (M7, a Phase I metabolite), and glycosylated FBZ (M8, a Phase II metabolite). The relative amounts of individual metabolites in roots and leaves of in vitro cultivated plants are shown in Table 1, and in different organs of whole soybean plant growing in greenhouse are shown in Table 2. The time-dependent formation of FBZ main metabolites (M7, M8) in soybean roots and leaves is given in Figure 2.

### 2.2. The Effect of FBZ on Isoflavones Content in Soybeans

**Figure 3:**
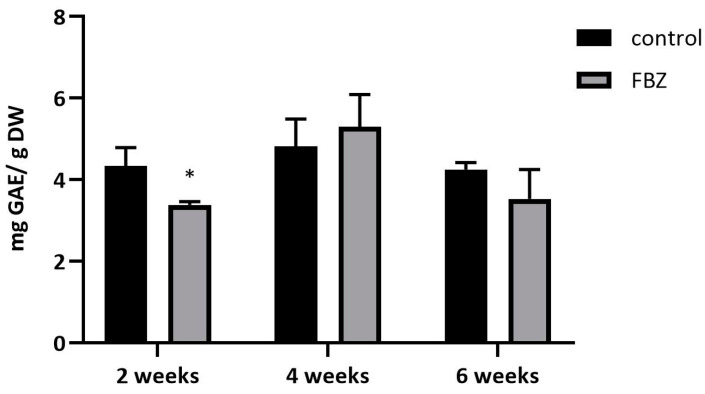
The time-dependent concentration of total phenolic compounds in soybean beans from control and FBZ treated plants. The total phenol content was expressed as milligrams of gallic acid equivalents per gram of dry weight extract (mg GAE/g DW). Mean ± SD, n = 6, * significant difference between FBZ treated and control plants.

FBZ was absorbed by soybean roots and transported to the leaves, pods, and beans. During this time, FBZ was partly metabolized and both the parent drug and its metabolites could affect the plant’s endogenous metabolism. Glycosylation represents the major FBZ biotransformation reaction in soybean as well as in other plants [11,21]. However, glycosylation is also an important reaction in the biosynthesis of plant polyphenols, and FBZ could interfere with this pathway. Moreover, the synthesis of plant polyphenols reacts extremely sensitively to the presence of various stress factors (including pharmaceuticals). Therefore, we hypothesized that FBZ could change the amounts of polyphenols in beans. During our experiment, the total amount of polyphenols in the beans from the FBZ-exposed and the control plants was assayed spectrophotometrically (Figure 3). FBZ reduced the total polyphenol content in soybean beans after 2 weeks of exposition only. In longer time interval the changes were insignificant perhaps because plants have adapted to chemical stress.

**Figure 4:**
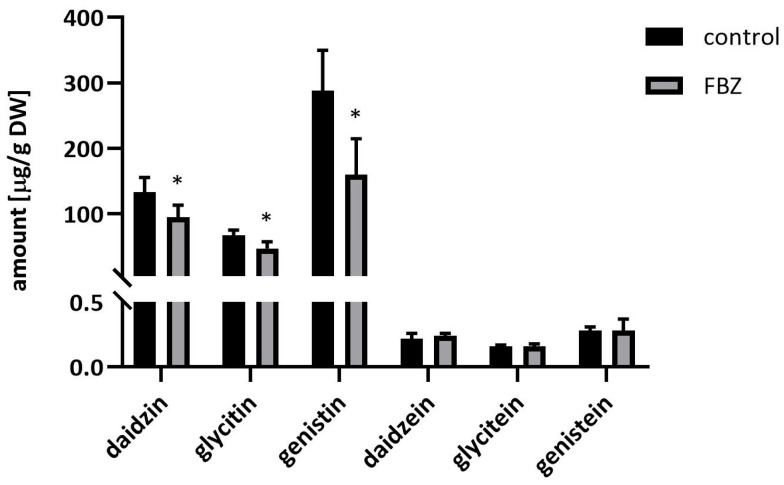
The concentration of isoflavones in control and FBZ treated soybean beans harvested after 42 days of the experiment. The final concentration of FBZ added to the soil was 12 µg/g dry soil. Mean ± SD, n = 6, * significant difference between FBZ treated and control plants.

Isoflavonoids play a vital role in plant defense response against various biotic and abiotic stresses [22], e.g., the changes in the amount and the composition of isoflavones in soybeans depends on the temperature during the vegetation period [23]. At the end of the experiment (6 weeks), we harvested the mature fruits and measured the concentration of individual polyphenols using HPLC. Along with the decrease of total polyphenol content, we observed an FBZ-mediated decrease in the ratio of glycosylated forms of isoflavones. The results presented in Figure 4 show that FBZ significantly decreased the content of glycosides of isoflavones (daidzin, glycitin, and genistin) but not the aglycones (daidzein, glycitein, and genistein). A possible explanation might be that the synthesis of glycosylated isoflavones interferes with the synthesis of glycosylated FBZ metabolites, as glycosylation is the most common biotransformation reaction of FBZ in soybean.

**Figure 5:**
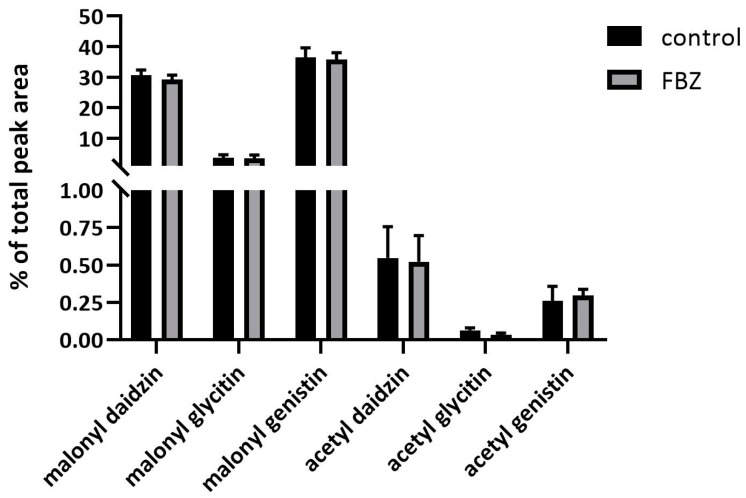
The concentration of malonylated and acetylated forms of daidzin, glycitin and genistin in control and FBZ treated soybean beans harvested after 42 days of the experiment. Mean ± SD, n = 6.

The exposure of soybeans to FBZ did not change the concentrations of malonyl daidzin, malonyl glycitin, and malonyl genistin (see Figure 5). Acetylated isoflavones were also identified, although their concentrations were only slightly above the detection limit, and changes between the exposed and control plants were not observed as well.

### 2.3. The Effect of FBZ on Antioxidant Enzyme Activities in Soybeans

Generation of reactive oxygen species (ROS) is one of the most abundant response of plant cell to the xenobiotic entrance. With the aim to protect their biomolecules from oxidative damage, plants have developed sophisticated antioxidant mechanisms. For example, the activation of antioxidant enzymes was observed in wheat seedlings cultivated on medium supplemented with the mixture of fluoroquinolone antibiotics [24]. The increase of antioxidant enzymes activity in the ribwort plantain was also observed under FBZ treatment [11].

**Figure 6:**
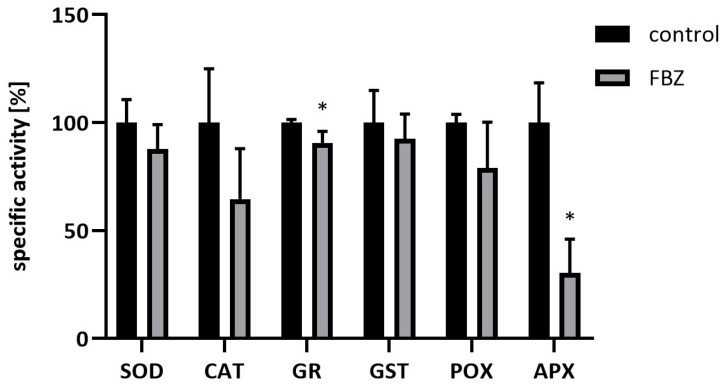
Specific activities of antioxidant enzymes (SOD, CAT, GR, GST, POX, and APX) in soybean beans after exposure to 10 µM FBZ for 42 days. Data are presented as the percentage of specific enzyme activity of control plants (100%). Mean ± SD, n = 6, * significant difference between FBZ treated and control plants.

Surprisingly, in our study, we observed that in the beans, FBZ decreased the activities of antioxidant enzymes (SOD, CAT, GR, GST, POX, and APX), while we established the presence of FBZ and its metabolites in beans. A significant decrease was found in GR and APX specific activities, other activities were decreased as well, but insignificantly (Figure 6).

Helminth control depends on extensive use of a limited range of anthelmintic drugs. For many species, this is predominantly a single class of drugs, which is likely to impose a strong selection pressure for drug-resistance spread in a number of parasites of livestock and domesticated animals. Resistance arises especially during underdosing when the parasites encounter a low concentration of an anthelmintic.

A very limited number of drugs are available for the treatment of some zoonotic helminth infections in humans. Soil-transmitted helminthiasis is usually treated with benzimidazole drugs mebendazole and albendazole, but there is a risk of developing resistance to treatment. Therefore, a clinical need for alternative anthelmintics becomes apparent. The possible transition of benzimidazoles from veterinary to human use was considered an appropriate strategy. An example of such a drug is oxfendazole, FBZ S-oxide, which has the potential to be an effective therapy for neurocysticercosis or echinococcosis [3,25,26].

The presence of low concentrations of FBZ and its metabolites in soybeans consumed by infected livestock or people may promote drug-resistance development since the parasites encounter a sub-lethal dose of the drug. This can be a problem, especially since as the use of meat and bone meals in livestock and poultry feeds decreases, and soy flour is more used due to its high protein content (beans contain about 36–40% crude protein and 18–21% oil).

Still, our most important finding is that FBZ and its hydroxylated metabolite M7 also occur in soybean beans, i.e., in the food-relevant part of the plant. Therefore, this anthelmintic drug can indeed find its way into human food and animal feed.

## 3. Materials and Methods

### 3.1. In Vitro Cultivation of Soybean in Seedlings

The soybean seeds [*Glycine max* (L.) Merr. cv, Moravia] were obtained from AGRITEC Research, Breeding and Services, Ltd., Šumperk, Czech Republic. The seeds had been sterilized in 70% ethanol for 1 min and next by 1% sodium hypochlorite supplemented by 0.02% detergent TWEEN 20 for a period of 10 min. and germinated on the agar MS medium [27] at 25 °C, with a 16-h photoperiod at 72 µmol of photons/m^2^/s. After 4 weeks the plantlets were replanted into Magenta boxes with the fresh MS medium supplemented by FBZ (final concentration 10 µM, pre-dissolved in DMSO) or by only DMSO in the corresponding concentration. The plants were harvested after 1 week.

### 3.2. Cultivation of Soybean Plants in a Greenhouse

The soybean seeds were obtained from AGRITEC Research, Breeding and Services, Ltd. The plants were grown in a greenhouse (23 °C, relative humidity about 60%) on soil supplemented by nitrazon (*Rhizobium* mix; Farma Žiro, s.r.o., Nehvizdy, Czech Republic) and regularly watered by tap water.

The time dependence (2, 4, and 6 weeks) of FBZ uptake was monitored. Six pots with soybean plants for each time interval were irrigated 3-times a week with a 10 µM solution of FBZ (pre-dissolved in DMSO) in tap water. The 6 control plants were watered by tap water supplemented by DMSO in the same concentration as the experimental plants. After 2, 4, and 6 weeks the roots, leaves and pods with beans were harvested, and the content of FBZ and its metabolites were determined. In mature beans, the contents of polyphenols and the activities of antioxidant enzymes were measured in FBZ treated and control plants. At the end of the experiment, the final amount of FBZ added in the soil was 5.681 mg per pot (12 µg FBZ/g dry soil).

### 3.3. HPLC Analysis of Isoflavones

For an analysis of isoflavones (daidzin, daidzein, genistin, genistein, glycitin, and glycitein) the mature beans from both, control and FBZ treated plants were used. The dry beans were ground in a laboratory mill and extracted with chilled 80% methanol (100 mg/mL) for 24 h by shaking. Extraction in an ultrasonic bath (3 × 30 min) was also included. The extracts were centrifuged (4000× *g*, for 15 min at 24 °C) and filtered (0.2 µm) before the HPLC analyses [28,29,30].

The content of daidzin, daidzein, genistin, genistein, glycitin, and glycitein was determined using HPLC (Alliance HPLC System, Waters, Santa Clara, CA, USA) equipped with a reverse phase SiC_18_Biospher packed stainless steel column (250 × 4 mm) and UV-vis detector (Waters 2998). The binary mobile phases were composed of water and acetonitrile with 0.1% acetic acid. In the mobile phase, a binary system was applied consisting of A (water) and B (acetonitrile) with the gradient from 85:15 (A:B, vol.%) to 65:35 (A:B, vol.%) in 50 min. The flow rate was 1 mL/min and the injection volume 20 µL. The substances were detected by comparing spectra and retention times of the samples along with standards (Sigma-Aldrich, Prague, Czech Republic) using a PDA detector. The content of the identified compounds was calculated from peak areas using external calibration at 254 nm.

### 3.4. UHPLC-MS/MS Analysis of FBZ and Metabolites

Fifty mg of each sample (soybean roots, leaves, pods, and beans) was extracted and analyzed. The extraction and analytical method for UHPLC-MS/MS analysis of FBZ and its metabolites (composition of the mobile phase and the setting of tuning parameters) have been described previously by Stuchlíková et al. [11].

The LC-MS analysis of isoflavone compounds was performed on UHPLC (Nexera, Shimadzu, Kyoto, Japan) liquid chromatography system coupled with QqQ mass spectrometer (LC-MS 8030, Shimadzu, Kyoto, Japan). Chromatographic separation was carried out on a Zorbax RRHD Eclipse plus C18 column (150 × 2.1 mm, particle sizes 1.8 μm, Agilent Technologies, Waldbronn, Germany). The column oven temperature was set at 40 °C, the mobile phase flow rate at 0.4 mL/min, and the sample injection volume was 0.1 µL. Analytes were separated using gradient elution consisting of water (A) and acetonitrile (B), both with an additional 0.1% (*v*/*v*) formic acid. The linear gradient was set as follows: 0 min-10% B, 2.2 min-30% B, 4 min-50% B, 6 min-100% B with an isocratic hold for 1.30 min followed with equilibration of the system for 3 min. The mass spectrometer ESI ion source parameters were set as follows: capillary voltage in positive ionization mode 4.5 kV, heat block temperature 400 °C, DL line temperature 250 °C, desolvation gas flow rate 12 L/min and nebulizer gas flow rate 3 L/min. Naringenin (5 µM) was chosen as an internal standard. The mass spectrometer was operated in MRM mode by monitoring selected transitions. All data were acquired using LabSolution LCMS software ver. 5.93 (Shimadzu, Japan).

### 3.5. Total Phenol Content Assay

The concentration of total phenolic compounds was determined using a modified colorimetric method of Folin–Ciocalteu [31]. Briefly, 100 µL of extract, blank (DMSO) or standard (gallic acid) was mixed with 25 µL of Folin–Ciocalteu reagent in a 96-well microplate and samples were shaken at 200 rpm at room temperature for 10 min. Then 75 µL of sodium carbonate (12% *w*/*v*) was added and samples were incubated in the dark at 37 °C for 1 h. The absorbance was measured using a microplate reader Tecan Infinite M200 (Tecan Group Ltd., Switzerland) at 760 nm. The calculation of the phenolic content was based on a calibration curve obtained with gallic acid and expressed as milligrams of gallic acid equivalents per gram of dry weight extract (mg GAE/g DW).

### 3.6. Antioxidant Enzymes Activity Assay

The freshly harvested beans were homogenized in dry ice, extracted in extraction buffer (50 mM KH_2_PO_4_; pH 7, 0.1 mM EDTA, 1% PVP K 30, 0.5% Triton-X 100), and centrifuged (20,000× *g*, 20 min, 4 °C). The supernatant was used for the subsequent enzyme assays. Protein content in the supernatant was determined according to [32]. The activities of superoxide dismutase (SOD), catalase (CAT), glutathione S-transferase (GST), glutathione reductase (GR), ascorbate peroxidase (APX), and peroxidase (POX) were measured spectrophotometrically using the common methods [11].

The specific activities of SOD CAT, GR, GST, POX, and APX were calculated per protein unit and expressed as percentages of the respective specific activities in the control plants.

### 3.7. Statistical Analysis

To determine changes in the treated plants, the data were processed using software STATISTICA.CZ version 12.0 (StatSoft, Prague, Czech Republic) or GraphPad Prism 9.0.2 (GraphPad Software, San Diego, CA, USA). Data of treated versus control plants were analyzed. Data are presented as mean values ± standard deviation, with significance set at *p* ≤ 0.05 according to one-factor ANOVA (specifically Tukey’s test). Significant differences were marked with an asterisk in the graphs.

## 4. Conclusions

In conclusion, anthelmintic drug FBZ is easily uptaken by soybeans and metabolized to many metabolites (16 was identified). The presence of parent compound and/or its metabolites in different plant organs can affect plant metabolism, and what is important, also the production of polyphenols, which represent pharmaceutically important compounds. In addition, the presence of FBZ in beans represent a significant risk of this drug entering animal feed and food consumed by humans, which could lead to promotion of the development of drug-resistance of helminths.

## Figures and Tables

**Table 1:** The relative amount (ratio between peak area of metabolite and peak area of internal standard) of individual FBZ metabolites in roots and leaves of in vitro soybean plantlets after one-week incubation with FBZ.

Metabolite Designation	Roots	Leaves
M1	41 ± 8	-
M2	8 ± 1	-
M3	9 ± 3	-
M4	109 ± 22	
M5	185 ± 40	-
M6	2 ± 0.02	-
M7	20,845 ± 4374	-
M8	-	6559 ± 2171
M9	232 ± 71	-
M10	10 ± 6	1047 ± 50
M11	14 ± 3	-
M12	19 ± 4	-
M13	69 ± 17	-
M14	200 ± 42	-
M15	83 ± 36	-
M16	1767 ± 648	-
FBZ	68,320 ± 14,998	141 ± 71

**Table 2:** The relative amount (ratio between peak area of metabolite and peak area of internal standard) of individual FBZ metabolites in roots, leaves, pods, and beans in soybean plants in different time intervals (2, 4, and 6 weeks) of treatment with FBZ.

	M7	M8	M16	FBZ
roots				
2 weeks	539 ± 39		22 ± 4	1165 ± 6
4 weeks	1013 ± 67		16 ± 13	1830 ± 67
6 weeks	636 ± 101		49 ± 36	2911 ± 579
leaves				
2 weeks	27 ± 5	800 ± 566		15 ± 6
4 weeks	87 ± 12	483 ± 387		28 ± 11
6 weeks	53 ± 20	3057 ± 690		60 ± 23
pods				
2 weeks	0.104 ± 0.006	1.34 ± 0.27		0.471 ± 0.053
4 weeks	0.320 ± 0.075	3.87 ± 0.21		0.212 ± 0.031
6 weeks	0.581 ± 0.023	1.06 ± 0.36		0.477 ± 0.217
beans				
2 weeks				0.304 ± 0.007
4 weeks	0.551 ± 0.408			0.486 ± 0.104
6 weeks	0.552 ± 0.418			1.686 ± 1.319

## Data Availability

The data presented in this study are available on request from the corresponding author.

## Supplementary Materials

The following are available online at https://www.mdpi.com/article/10.3390/ijms22136647/s1, Table S1: Biotransformation of FBZ in soybean–main peaks detected by UHPLC-MS/MS. Retention time, theoretical molecular weight, molecular formula and selected SRM transitions.
