# Supplementary material for: Soybean (Glycine max) Is Able to Absorb, Metabolize and Accumulate Fenbendazole in All Organs Including Beans
Source: Int J Mol Sci. 2021 Jun 22;22(13):6647. doi: 10.3390/ijms22136647 (PMC8268216; doi:10.3390/ijms22136647)
Supplement: Supplementary file 1 [file ijms-22-06647-s001.zip › ijms-1262026-supplementary.pdf]

Table S1. Biotransformation of FBZ in soybean – main peaks detected by UHPLC-MS/MS. Retention time, theoretical molecular weight, molecular formula and selected SRM transitions.

t_R [min]	Theoretical *m/z* Values of $[M+H]^+$ ions	Elemental Composition	Description of metabolite formation		Product Ions of $[M+H]^+$, *m/z*	Metabolite Designation
			Phase I	Phase II		
2.631	478.1278	$C_{21}H_{23}N_3O_8S$	2*(+O), hydrolysis	Glycosidation, *O*-acetylation	258, 133	M1
2.842	478.1278	$C_{21}H_{23}N_3O_8S$	2*(+O), hydrolysis	Glycosidation, *O*-acetylation	258, 133	M2
3.090	478.1278	$C_{21}H_{23}N_3O_8S$	2*(+O), hydrolysis	Glycosidation, *O*-acetylation	258, 133	M3
3.504	478.1278	$C_{21}H_{23}N_3O_8S$	2*(+O), hydrolysis	Glycosidation, *O*-acetylation	258, 133	M4
3.942	478.1278	$C_{21}H_{23}N_3O_8S$	+O	*N*-glycosidation	316, 284, 191	M5
4.031	478.1278	$C_{21}H_{23}N_3O_8S$	2*(+O), hydrolysis	Glycosidation, *O*-acetylation	258, 133	M6
5.202	316.075	$C_{15}H_{13}N_3O_3S$	Hydroxylation	-	266, 207	M7
6.086	462.1329	$C_{21}H_{23}N_3O_7S$	-	*N*-glycosidation	300	M8
6.492	462.1329	$C_{21}H_{23}N_3O_7S$	+O, hydrolysis	Glycosidation, *O*-acetylation	242	M9
6.567	462.1329	$C_{21}H_{23}N_3O_7S$	-	*N*-glycosidation	300	M10
6.683	462.1329	$C_{21}H_{23}N_3O_7S$	+O, hydrolysis	Glycosidation, *O*-acetylation	242	M11
6.891	462.1329	$C_{21}H_{23}N_3O_7S$	+O, hydrolysis	Glycosidation, *O*-acetylation	242	M12
6.851	462.1329	$C_{21}H_{23}N_3O_7S$	-	*N*-glycosidation	300, 268	M13
7.256	462.1329	$C_{21}H_{23}N_3O_7S$	+O, hydrolysis	Glycosidation, *O*-acetylation	242	M14
7.605	462.1329	$C_{21}H_{23}N_3O_7S$	-	*N*-glycosidation	300, 268	M15
8.08	462.1329	$C_{21}H_{23}N_3O_7S$	-	*N*-glycosidation	300, 268	M16
9.61	300.0801	$C_{15}H_{13}N_3O_2S$	-	-	268, 159, 131	FBZ
